# Neurophysiological Verbal Working Memory Patterns in Children: Searching for a Benchmark of Modality Differences in Audio/Video Stimuli Processing

**DOI:** 10.1155/2021/4158580

**Published:** 2021-12-20

**Authors:** Bianca Maria Serena Inguscio, Giulia Cartocci, Nicolina Sciaraffa, Claudia Nasta, Andrea Giorgi, Maria Nicastri, Ilaria Giallini, Antonio Greco, Fabio Babiloni, Patrizia Mancini

**Affiliations:** ^1^Department of Sense Organs, Sapienza University of Rome, Viale dell'Università 31, 00161 Rome, Italy; ^2^BrainSigns Srl, Lungotevere Michelangelo, 9, 00192 Rome, Italy; ^3^Department of Molecular Medicine, Sapienza University of Rome, Viale Regina Elena 291, 00161 Rome, Italy; ^4^Department of Computer Science, Hangzhou Dianzi University, Xiasha Higher Education Zone, 310018 Hangzhou, China

## Abstract

Exploration of specific brain areas involved in verbal working memory (VWM) is a powerful but not widely used tool for the study of different sensory modalities, especially in children. In this study, for the first time, we used electroencephalography (EEG) to investigate neurophysiological similarities and differences in response to the same verbal stimuli, expressed in the auditory and visual modality during the n-back task with varying memory load in children. Since VWM plays an important role in learning ability, we wanted to investigate whether children elaborated the verbal input from auditory and visual stimuli through the same neural patterns and if performance varies depending on the sensory modality. Performance in terms of reaction times was better in visual than auditory modality (*p* = 0.008) and worse as memory load increased regardless of the modality (*p* < 0.001). EEG activation was proportionally influenced by task level and was evidenced in theta band over the prefrontal cortex (*p* = 0.021), along the midline (*p* = 0.003), and on the left hemisphere (*p* = 0.003). Differences in the effects of the two modalities were seen only in gamma band in the parietal cortices (*p* = 0.009). The values of a brainwave-based engagement index, innovatively used here to test children in a dual-modality VWM paradigm, varied depending on n-back task level (*p* = 0.001) and negatively correlated (*p* = 0.002) with performance, suggesting its computational effectiveness in detecting changes in mental state during memory tasks involving children. Overall, our findings suggest that auditory and visual VWM involved the same brain cortical areas (frontal, parietal, occipital, and midline) and that the significant differences in cortical activation in theta band were more related to memory load than sensory modality, suggesting that VWM function in the child's brain involves a cross-modal processing pattern.

## 1. Introduction

The term working memory (WM) [1] refers to the type of memory that is active and relevant for short periods of time, usually only seconds [2]. Specifically, it is the theoretical construct used in cognitive neurosciences to refer to the system or mechanism underlying the maintenance of relevant information during cognitive task performances [3, 4]. Baddeley-Hitch's WM model proposes a tripartite system organized in a central executive and two subsidiary systems: the phonological loop, capable of holding verbal information, and the visuospatial sketchpad, which exercises a parallel function for spatial information [1, 5].

Although the WM multicomponent model is influential in scientific thinking, its neural basis remains poorly specified [6].

There is evidence that WM provides a mental workspace used in many fundamental learning activities during lifespan, including *literacy* [7, 8], *reading* [9], and *numeracy* [10]. These findings have important implications in education, particularly for children with neurodevelopmental disorders and sensory deficits [11].

N-back task [12] has become a prototypical measure in functional neuroimaging studies that allows identification of the neural mechanisms supporting WM [13] (see [14] for a meta-analysis). In fact, studies consistently find that n-back performance is associated with activation in prefrontal and parietal cortical regions widely recognized as the primary neural substrates that underlie working memory processes [2, 14–18] and in particular visual and auditory stimuli processing [19, 20]. Moreover, patterns of neural activation associated with n-back performance have been shown to vary with the type of information held in working memory (e.g., verbal or spatial), as well as task difficulty (i.e., 0-, 1-, and 2-back) (see [21, 22] for review).

It has been suggested that the prefrontal cortex (PFC) is critical for resilient information maintenance during WM tasks [23]. Given its functional connections with the posterior parietal cortex, Dorsolateral PFC plays a crucial role in both verbal and visuospatial WM [14, 24] (see [25] for review). Moreover, stronger frontoparietal synaptic connectivity may be one of the mechanisms involved in WM capacity development during childhood [26]. Investigators have mapped WM-related activity to sensory association cortices and PFC and some regions show specificity to sensory stimuli modality (see [27, 28] for review).

Adults have been demonstrated to have functional hemispheric specialization for WM with a refinement of verbal processing operated by the left hemisphere, whereas the right hemisphere appears more specialized in visuospatial processing [22, 29–31]. Few studies have examined this potential sensorial input dissociation or whether more distinct or lateralized patterns of brain responses and considered signature of WM emerge across development [32]. In fact, brain structures and neural processes subserving WM continue to develop during childhood [33, 34], and it is known that changes in PFC are related to cognitive development achievements occurring during childhood as well [35–37].

Neuroimaging assessments for verbal [38] and visuospatial [39–41] stimuli support the evidence that WM-related activation is greater and more widely distributed in the child brain than the adult brain [33, 42]. This may reflect ongoing maturation and synaptic fine tuning during development [37].

EEG neuroimaging studies, principally in adult populations, have evidenced enhanced activity during WM load-specific modulations by different bands, in particular theta, alpha, and gamma in numerous brain areas [43–47]. Moreover, a brainwave-based mental engagement index (EI) previously defined by Pope and colleagues [48] as

(1) $$\begin{array}{c} \text{EI}=\frac{\text{Beta}}{\text{Alpha}+\text{Theta}}, \end{array}$$

has proven to be successful in distinguishing brain attentive states and to correlate with emotions and mental workload in memory tasks [49–52]. Furthermore, McMahan and colleagues [53], comparing EI with other EEG engagement indexes (frontal theta, ratio of frontal theta to parietal alpha), found the ratio between beta and the sum of alpha and theta to be the best algorithm for calculating the engagement levels of players playing video games. Research has shown that classifiers using physiological features are able to determine the level of cognitive activity in tasks with a high level of accuracy [54]. However, studies that have applied EI to assess children's cognitive engagement are rare (see [55, 56]), and to the best of our knowledge, there are no published EEG studies involving the assessment of WM through the n-back task in children younger than 13.

To date, there have not been many investigations on the specific relationship between cognitive and neurophysiological developmental changes in WM functions during childhood [57–60]. Furthermore, several studies focus on the visuospatial component of WM processing (e.g., [61–63]). Better understanding of the development of WM functions would help in the determination of what is normal and what is pathological at different ages and in the development of new learning, teaching, and cognitive training strategies [64–69].

Verbal WM (VWM) is a specific human form of WM that appears to play a significant role in language comprehension and problem solving [70]. It is particularly important given the role that linguistic processes play in the higher-cognitive processes [18]. Most of our knowledge of the neural network underlying VWM is based on studies using visually presented stimuli [22, 39, 71]. There have been few reports of investigations on the purely neural basis of auditory VWM [72–74], and even fewer have directly examined modality differences using similar tasks in a within-subjects design [6]. Specifically, with the aim of digging into the neural mechanisms underlying the model of processing of the verbal components of WM (e.g., phonological loop [75]), only four neuroimaging studies concerning adult populations have considered the effect of n-back task modality on brain activation [6, 76–78]. Those studies reported contrasting findings and employed neuroimaging techniques different from EEG, which we selected for its high temporal resolution. It is noteworthy that most of the published studies on working memory involve the use of brain-imaging techniques that are more invasive and less ecological than EEG, such as fMRI (e.g., [63, 79–81]) and PET (e.g., [82]) that are often impractical for use on children [59, 83]. Precisely, no studies have used EEG to assess neural responses during verbal n-back tasks with different sensory stimuli (visual and auditory), in particular in healthy children.

The aim of this study is to examine EEG activation during VWM processing of *auditory* and *visual* stimuli presented to children during n-back task performance [12]. The distinction between auditory VWM and visual VWM is important, with implications for both theoretical and experimental research on the neural processes underlying WM. In fact, as Crottaz-Herbette and colleagues [6] pointed out, elucidation of similarities and differences in the processing of different types of stimuli can provide insight into the internal representations of stimuli in WM. Indeed, to date, the considerable theoretical debate on WM features is evident in the many cognitive studies (e.g., [84–86]) that investigate whether WM storage is mediated by distinct subsystems for auditory and visual stimuli [5] or by a single central capacity system [87].

Moreover, the discrepancies in the studies regarding adults reported above, on the assumption of an a-modal VWM system [6, 76–78] and the absence of studies on healthy and clinical child populations, evidence a scientific void that must be filled. In an attempt to tackle this issue, the experimental investigation of the neural underpinnings of auditory and visual stimuli processing and the consideration of a possible cross-modal activation during childhood in a VWM task appears extremely important for the evaluation of healthy development of children with or without sensory impairment.

We hypothesized that, in children, the involvement of the theoretical phonological loop [75], which underpins verbal WM processing, is neurally mediated indifferently by both auditory and visual stimuli. Thus, with both visual and auditory-verbal WM n-back tasks, we expected to find the following:

1. There are no differences in EEG activation patterns in the cortical areas involved in VWM function in response to the two different sensory stimuli.
2. As largely confirmed by the literature on adults presented above, significant differences in EEG activation in response to auditory and visual stimuli depend only on memory load variations (0-1-2-back) and the EEG findings correlate with behavioral results also in children.

Confirmation of our hypotheses could indicate the possibility of identifying a neurophysiological benchmark of auditory-visual VWM in healthy young individuals, also allowing further comparisons with clinical research groups.

## 2. Material and Methods

### 2.1. Participants

Thirteen right-handed children aged 7–13 years (for age and sample size definition (children were selected according to previous studies), see [88, 89]) were enrolled in the study. Two participants were subsequently excluded because of the lack of cooperation in the task training accomplishment. Therefore, the final experimental sample was composed of 11 children (6 M and 5 F; mean age = 10.83 ± 1.87 yr).

Prior to the experiment, participants and their parents were informed about the study. We obtained informed written consent from the parents and verbal assent from the children. Participation in the study was voluntary; participants did not receive compensation for taking part. The experiment was conducted according to the principles outlined in the Helsinki Declaration of 1975, revised in 2000, and approved by the Institutional Ethics Committee of Policlinico Umberto I- Rome (no. 259/2020).

Subject selection was based on diagnostic screening using the *Peabody Picture Vocabulary Test-III* [90], a standardized measure of receptive oral vocabulary, and *Raven's Standard Progressive Matrices* [91], a standardized test of nonverbal spatial reasoning. Both tests use standardized scoring based on participant age (*µ* = 100, SD = 15). Exclusion criteria for enrollment in the study were left-handed children, due to past evidence of handedness influence on cerebral laterality [92]; children with scores below the standard average for their age (taken from test norms) on PPVT and RPM; and those diagnosed with neuropsychiatric disorders and/or sensorial deficits.

### 2.2. Experimental Design and Procedure

Participants performed two verbal n-back tasks [12] with varying memory load from 0-back to 2-back during EEG recording: (i) an auditory n-back task (AUD-task) in which stimuli were presented aurally and (ii) a visual n-back task (VIS-task) in which stimuli were presented visually on a computer screen.

Task administration order was randomized across participants. Therefore, approximately half of the participants started with the AUD-task and the second half with VIS-task. In addition, the order of presentation of the n-back blocks was randomized across participants; in other words, it did not follow an increasing level order.

**Figure 1:**
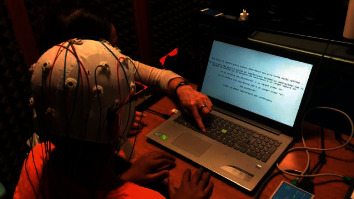
Explanation of task execution before training section. The picture shows a representation of the detailed explanation of the task to each participant before the effective measurement session.

**Figure 2:**
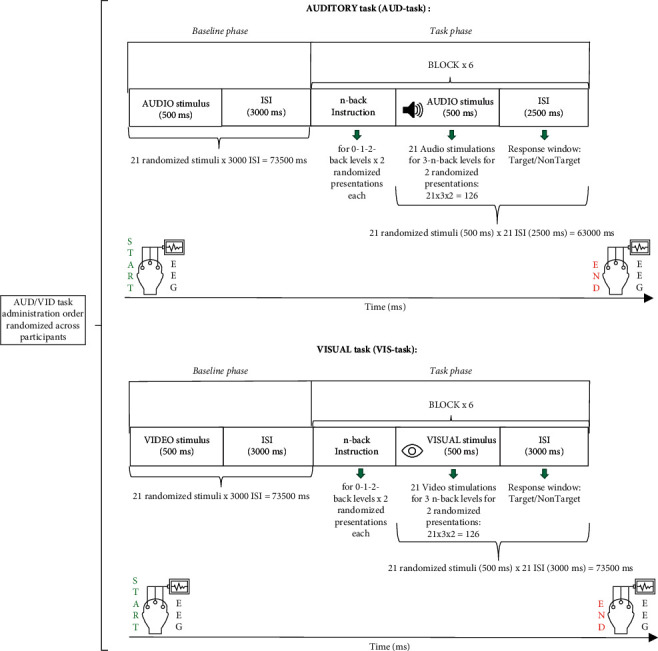
Experimental design with trial timeline. Schematic illustration for each of the two n-back tasks (auditory and visual modalities) performed by subjects during electroencephalography (EEG) recording. Each modality task started with the Baseline phase followed by the Task phase.

**Figure 3:**
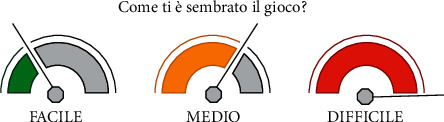
Illustration of perceived difficulty. Each participant was asked to indicate through this image a level of perceived difficulty after each visual and auditory task. *Note*. Translation of the Italian text: *Come ti è sembrato il gioco?* *=* How was the game?; *Facile* = easy; *Medio* = medium; *Difficile* = hard.

 
*Stimuli*: verbal material consisted of auditory and visual stimuli referring to seven consonants (c, g, k, p, q, t, and v), already used and described in previous studies [93–96]. Vowels were excluded in order to reduce the likeliness of participants developing chunking strategies, as suggested in Grimes et al. [97]. Stimuli exposure pretest was performed to ensure correct perception by participants. Auditory stimuli, lasting 500 ms and presented with an interstimulus interval (ISI) of 2500 ms [96], were spoken by a female voice set at 65 dB SPL intensity, in order to ensure comfortable audibility, transmitted by two audio speakers placed at face level 1 meter in front of the participant. Visual stimuli (duration 500 ms; ISI 3000 ms) [88] consisted of the same seven consonants (Consolas font-130) presented one at a time on a grey background in the center of a monitor screen placed at eye level, 50 cm distant from the participant.
  Task execution: participants had to respond in the ISI just after the presentation of each letter by pressing a button (D/K) to indicate whether the letter was a target (K) or a nontarget (D); thus, there was a behavioral response in either case. In the 0-back condition, the letter X was the target. In the 1-back condition, a letter was a target when it was the same as the one presented immediately before. In the 2-back condition, a letter was a target when it was the same as the one that presented two letters before. Participants were given detailed instructions for proper task performance and a training session before the effective measurement session in order to familiarize them with the experimental procedures (Figure 1).
  Task structure: the three WM load levels (0-1-2-back) were presented in six blocks (2 for each level) for each task (auditory and visual). The blocks were constituted by 21 randomized stimuli (30% target) [88]. At the beginning of each modality task, there was a *Baseline phase*, during which subjects were asked to remain relaxed, with no task except to look at the screen while auditory or visual stimuli were presented. During the *Baseline phase,* the 7 stimuli were repeated randomly 3 times (duration 500 ms with 3000 ms ISI), creating a 21 item block, analogous to the experimental blocks. Subsequently, the *Task phase* consisted of two randomized presentations of each of the three blocks, which began. Thus, every single session consisted of 3 n-back levels per 2 presentations, for a total of 6 blocks in randomized order for both audio and video tasks (Figure 2). Half of the participants started with the visual stimuli task and the other half with the auditory task.
  A Lenovo PC (monitor resolution 1024 × 768) displayed and controlled stimuli presentation and participant responses (reaction times (RTs); correct responses (CRs)) through the software package E-Prime (Psychology Software Tools, Pittsburgh, Pa, Version 3.0).
  Procedure: the participant was seated on a comfortable chair in an audiometric test room, and the experimental procedure was explained. In order to reduce muscular artifacts in the EEG signal, participants were instructed to assume a comfortable position and to avoid unnecessary movement. After each *Task phase*, participants indicated the perceived task difficulty (easy-medium-hard) on a stylized image (Figure 3). At the end of the entire experimental session, they were asked to evaluate which of the two tasks (visual or auditory) was the most difficult.

### 2.3. Behavioral Data Analysis

Performance was assessed in terms of accuracy (ACC) and RTs. ACC was calculated as the percentage of CRs for each task condition (each n-back level for both auditory and visual modality tasks); RTs were measured from the time of stimulus offset. In order to integrate these two aspects of performance, Inverse Efficiency Score (IES = RT/1 − PE) [98] was calculated, where RT is the subject's average RTs for correct answers (target/nontarget), and PE is the subject's proportion of errors for each condition. IES can be interpreted as the RT corrected for the number of errors committed [99].

### 2.4. EEG Recording and Data Analysis

EEG was recorded through a digital ambulatory monitoring system (BePlus System -EBNeuro, S.p.A., Italy) with a sampling frequency of 256 Hz. Twenty channels (Fpz, Fz, F3, F4, F7, F8, Cz, C3, C4, T7, T8, Pz, P3, P4, P7, P8, Cp5, Cp6, O1, and O2) were referred to the participants' earlobes, and impedance was kept below 10 kΩ. A 50 Hz notch filter was then applied to remove power interference. EEG signal was band-pass filtered with a 5th order Butterworth band-pass filter (1–45 Hz) to reject continuous components and high-frequency interferences like muscular artifacts. The Fpz channel was used to remove eye-blink contributions by the REBLINCA algorithm [100, 101] without losing data. Other artifacts were eliminated by specific procedures of the EEGLAB toolbox [102].

EEG dataset was segmented into epochs starting 500 ms before stimulus onset and ending 2500 ms after its offset. This temporal windowing was chosen to respect EEG stationarity and allow for a high number of observations compared to the number of variables considered in the analysis [103]. Three criteria were applied in order to identify artifacts according to published procedures [55, 104]. In particular, all the epochs exceeding the *threshold criterion* (±80 *µ*V) were marked as artifacts, as well as those that did not meet the *trend estimation criterion (*slope higher than 40 *µ*V/s or less than 0.3 *µ*V/s). The EEG epoch was also considered an artifact if the signal sample to sample difference (*sample to sample criterion)* in terms of absolute amplitude was higher than 30 mV, that is, when an abrupt variation (nonphysiological) occurred. Finally, epochs marked as artifact were removed from the EEG dataset such that all analyses were based on clean EEG signals [105–109]. Definition of EEG bands of interest involved the identification of subjective differences in terms of brain activity. Individual Alpha Frequency (IAF) in Hertz was computed on a 60-second long-closed eyes segment, recorded before the *Baseline phase* [110].

Each band was then defined as IAF ± *x* where *x* was an integer in the frequency domain [110]; thus, electrophysiological activity was divided by filtering EEG signals in the following frequency bands: theta (IAF – 6 ÷ IAF – 2 Hz), low alpha (IAF – 2 ÷ IAF Hz); upper alpha (IAF ÷ IAF + 2), alpha (IAF – 2 ÷ IAF + 2 Hz), beta (IAF + 2 ÷ IAF + 16 Hz), and gamma (IAF + 16 ÷ IAF + 30 Hz).

Then, the Power Spectral Density (PSD) [111] was calculated for each epoch and channel, using a Hanning window of 1 sec and an overlap of 500 ms. Cortical distribution of band modulation analysis was based on averages of the data for frontal, parietal, occipital, midline, and hemisphere electrode locations. The specific channels considered were frontal, F3, F4, F7, F8, and Fz; parietal, P4, P3, P7, and P8; occipital, O1 and O2; midline, Fz, Cz, and Pz; left hemisphere, F3, C3, T7, P3, and O1; and right hemisphere, F4, C4, T4, P4, and O2.

Moreover, EI [48] was calculated according to the formula specified above.

PSD data were normalized with respect to the baseline to limit influences on scores due to subjective stimuli perception on VWM EEG recording [112].

### 2.5. Statistical Analysis

The statistical analyses were conducted for neurophysiological and behavioral data, respectively. The Shapiro–Wilk normality test [113] was applied to the datasets under investigation. Then, depending on the results, parametric analysis of variance (ANOVA) or nonparametric ANOVA [114] was done. Both behavioral and neurophysiological values were entered in a 2 × 3 factorial ANOVA with 2 factors: factor modality (with two levels: *audio* and *video*) and factor load (with three levels: *0*-*1*-*2*). Duncan's post hoc test [115] was used to investigate statistically significant results of ANOVA tests; partial eta squared (*η*_*p*_^2^) effect sizes [116, 117] were reported. Finally, Pearson's correlation coefficient (*r*) [118] was used to assess the relationship between behavioral data and neurophysiological values. An alpha value (*α*) of 0.05 was used as the cutoff of significance [119].

## 3. Results

### 3.1. Behavioral Results

Behavioral results (RT, ACC, and IES) are presented in Tables 1 and 2.

**Figure 4:**
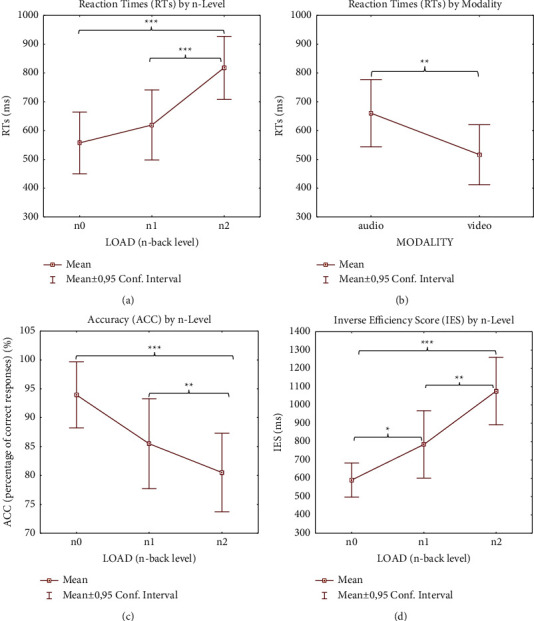
Behavioral results. Significantly different ANOVA (see Tables 1 and 2) behavioral results (RT (a) and (b); ACC (c); IES-4 (d)) according to load and modality factors. *Note*. Significant differences between load condition, modality condition, and load x modality condition emerging from the post hoc test are indicated (^*∗*^*p* ≤ 0.05; ^*∗∗*^*p* ≤ 0.01; ^*∗∗∗*^*p* ≤ 0.001).

RTs and IES increased, and ACC decreased with increasing memory load. Post hoc analysis showed that auditory modality produced significantly longer RTs than visual modality (*p* = 0.008) (Figure 4(b)) and showed a significant increase in RTs as the n-back level increased (*p* < 0.001) both between 0- and 2-back and between 1- and 2-back (Figure 4(a)) regardless of modality.

The overall ACC score percentages were greater during all auditory n-back levels (96.96, 87.60, and 87.01 for 0-1-2 levels, resp.) than for visual ones (90.90, 83.33, and 74.02 for 0-1-2 levels, resp.). Post hoc analysis showed significantly lower accuracy for both the 1-back and the 2-back compared to the 0-back level (*p* = 0.007 and *p* < 0.001, resp.), independently of modality (Figure 4(c)). This trend was reflected in the IES data: post hoc results showed significant differences between 0 and 2 and 1 load (*p* < 0.001 and *p* = 0.028 resp.) and between 1 and 2 loads (*p* = 0.002) (Figure 4(d)).

### 3.2. Neurophysiological Results

**Figure 5:**
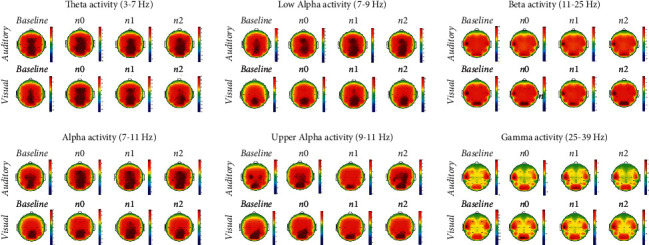
Topographical representation of visual and auditory-verbal working memory in the different frequencies of interest. Topoplots represent the average Power Spectral Density (PSD) of all eleven subjects in the 19 electrodes sites on the scalp for alpha, theta, beta, and gamma frequency bands during the Baseline phase and during each n-back task condition (n-level x modality). Colors describe high (warm-color coded) and low (cold-color coded) intensities of PSD (see color bars).

EEG activation during session recordings is shown in Figure 5; neurophysiological statistical results are shown in Table 3.

**Figure 6:**
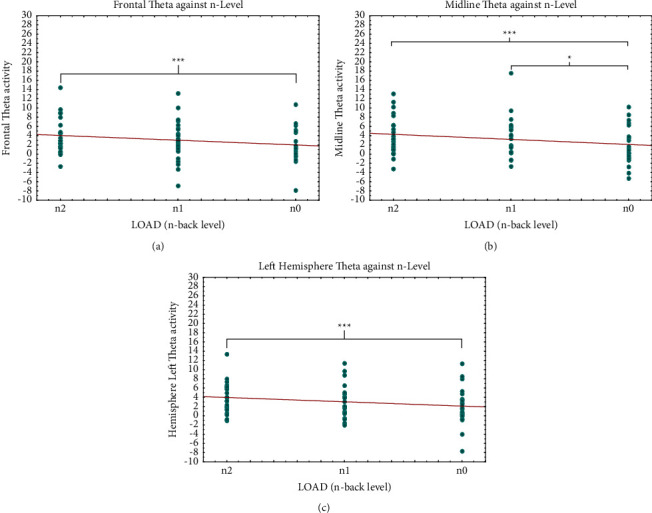
Theta band results. Significantly different ANOVA (see Table 3) neurophysiological theta results in different brain areas (resp., in frontal (a); midline (b); and left hemisphere (c) in relation to load. *Note*. Significant differences between load conditions emerging from the post hoc test are indicated (^*∗*^*p* ≤ 0.05; ^*∗∗*^*p* ≤ 0.01; ^*∗∗∗*^*p* ≤ 0.001).

Post hoc analysis regarding the frontal area revealed that theta power was higher in 2-back than in 0-back level tasks (*p* = 0.008) (Figure 6(a)). Similar increased theta band activity was also evidenced in the midline area, where post hoc analysis showed higher activation related to increasing n-back task difficulty (from 0-back to 1-back levels, *p* = 0.013; from 0-back to 2-back levels, *p* = 0.001) (Figure 6(b)).

**Figure 7:**
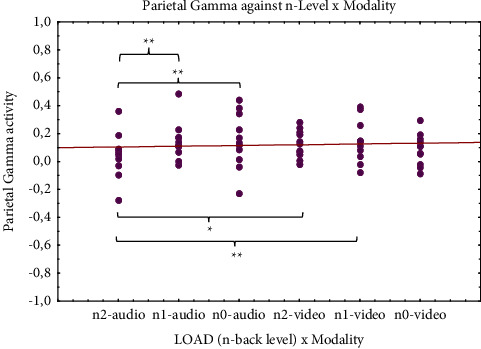
Gamma band results. Significantly different ANOVA (see Table 3) neurophysiological gamma results (parietal brain area) in relation to load and modality. *Note*. Significant differences between load x modality condition emerging from post hoc test are indicated (^*∗*^*p* ≤ 0.05; ^*∗∗*^*p* ≤ 0.01; ^*∗∗∗*^*p* ≤ 0.001).

The remarkable effect of load factor on theta band activity was also evidenced in the left hemisphere comparing 2- and 0-back levels (*p* = 0.001) by post hoc analysis (Figure 6(c)). The significant different activation on theta band did not depend on modality factor. Differently, post hoc analysis of gamma activity in the parietal area showed sensitivity to audio modality for each level of load × modality interaction, except for 0-back video condition (*p* = 0.165) (Figure 7). Specifically, gamma activation was lower during the 2-back level in the audio task than for the same level in the video task (*p* = 0.017) and also compared to 1-back level audio (*p* = 0.005) and video (*p* = 0.005) stimulation. The difference in gamma activation during the audio 2-back level condition was even more pronounced when compared to that seen with the 0-back level in audio presentation (*p* = 0.004).

**Figure 8:**
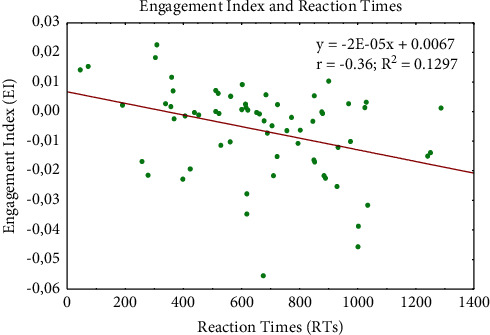
Engagement index and reaction times relationship. Scatter plot showing the negative correlation between engagement index and reaction times values.

Finally, the post hoc test evidenced increased EI values comparing both the 2-back and 0-back levels (*p* < 0.001) and the 2-back and 1-back levels (*p* = 0.031), regardless of modality. Moreover, a negative correlation was observed between EI values and reaction times (*r* (64) = –0.36; *p* = 0.002) (Figure 8).

## 4. Discussion

### 4.1. Performance

RTs were statistically significant regarding both load and modality factors, as reported in previous studies (i.e., [94]). Participants' responses were significantly slower during the hardest level (2-back) task than the medium (1-back) and simplest (0-back) levels and during auditory compared to visual tasks (Figures 4(a) and 4(b)). The latter finding conflicts with the hypothesis that auditory stimuli have more durable feature binding [120] and longer lasting representations and thus stimulate enhanced performance [121, 122]. However, there are exceptions to the finding that the auditory condition improves the speed of responses during WM tasks (i.e., [6, 123, 124]). A possible explanation, also advanced by Amon et al. [124], might be that visual stimuli were processed more quickly, but the accuracy scores (90.55% versus 82.33% for auditory and visual conditions, resp.) suggest a more accurate stimulus processing. Furthermore, longer RTs during the auditory condition (*µ* = 731.70 ± 262.794) reflected the subjects' perception of difficulty (54.54% of the participants perceived more difficulty with auditory than visual tasks). Another plausible interpretation, complementary to the previous one, could be that visual WM reaches functional maturity earlier than the corresponding auditory system [33]. Response accuracy (ACC) and IES worsened with increasing n-back levels, but the effect of memory load performance was generally more statistically evident in relation to RT than ACC and IES, possibly due to a ceiling effect [125] (Figures 4(c) and 4(d)).

### 4.2. Electroencephalographic Activation

Neural oscillations provide an effective measure to assess the underlying neural mechanism that enables and controls memory load and memory decay [126]. Previous reports on a quantitative comparison of neurophysiological patterns during different n-back tasks (e.g., [127–131]) involved mostly adult populations (see, e.g., the meta-analyses in [21, 132, 133] study); only rarely did they involve visual and auditory VWM, in particular in children. This study, instead, focused on cortical activation in children during auditory and visual n-back tasks.

As expected, we found that stimulation of VWM in children appears to activate generally the same brain regions as in adults (Figure 5), albeit in a more widely distributed pattern [38]. Thus, our investigation of EEG differences in stimuli processing during n-back tasks may be an important tool for understanding developing neural functioning.

We observed a significant increase in theta power in the frontal area related to memory load (Figure 6(a)). This observation is in line with the findings of an EEG study by Gevins et al. [43], reporting the relationship between the increase in frontal theta activity and the task difficulty in subjects performing an n-back task. One interpretation of this activation pattern can be that the increase reflects enhanced attention [45, 134, 135] or effortful cognitive processes [136, 137]. Moreover, multiple function neuroimaging studies have shown that some areas of the PFC are engaged in maintenance and recall of WM representation [14].

EEG evidence of human theta band activity is maximal on the scalp close to the frontal midline; it is often present during waking and is stronger on average during various types of demanding cognitive tasks [43, 138]. Significant theta power changes in the midline area (Figure 6(b)) under various conditions show that this pattern increases with memory load, in agreement with previous studies demonstrating that theta band power in frontal midline scalp increases with mental effort [45, 138–140]. We note that, in our investigation, there was no evidence that theta band activity in frontal and midline areas was influenced by modality (auditory or visual) (Figures 6(a) and 6(b)), results that appear to support our prediction of an a-modal processing of VWM.

The study included exploratory analysis to investigate possible hemispheric lateralization of auditory and visual VWM. It is known that children show hemispheric lateralization in the left frontal and temporal lobes during the VWM task [38] and greater activation of spatial WM in the right frontal, parietal, and occipital cortices [141]. Thus, there may be hemispheric asymmetry for verbal and spatial WM [22], but to date, there have been no reports of investigations on specific modality dissociation of VWM in children. Our results showed a significant strength of lateralization of theta activity in the left hemisphere related to increasing task difficulty but no significant variations related to different task modalities (Figure 6(c)). Lack of influence of audio or video modality on verbal WM is also supported by the absence of significant differences in activation on the F7 electrode that coincides with the Brodmann [142] areas 44–45 corresponding to the Broca [143] language area [144, 145]. The absence of differences in activation of this area related to modality, auditory or visual, could be an indication that VWM is processed as language regardless of stimulus modality in our sample.

Gamma band is another candidate for an EEG signature of WM load [146]. There is evidence that gamma oscillations are involved in perception [147, 148] and are thought to reflect processes related to activation and maintenance of neuronal object representations [149]. Data also suggest a role of gamma in WM as well as perception [150]. Several studies associate this band with higher-cognitive processes [151–154]. Studies have also shown that, in addition to perceptual processing, gamma band activity accompanies many other important cognitive functions such as attention [155–157], arousal [158], language perception [159], and object recognition [160].

Our results show decreasing gamma power in parietal areas to audio stimulation within n-levels (Figure 7). Indeed, the activation trend is inversely proportional to the audio task level, whereas no significant evidence is observed for the video task within n-levels. Comparison of the findings regarding responses to the two different sensory modalities leads us to hypothesize that gamma activity in the parietal cortex is the strongest during the simplest audio condition (0-back) and decreases in the most complex task (2-back). The stronger activation observed during the auditory task (but not visual) seems to be the opposite of the findings of an fMRI study [6]. However, this latter study differed from ours in relation to both the neuroimaging technique used as well as the experimental sample (adults instead of children) and the type of stimuli administered. On the other hand, our finding partially agrees with those of other studies that report enhanced unisensory auditory gamma band activity [161, 162]. Thus, the differences found in parietal areas seem to be of “sensorial origin” rather than strictly connected to the cognitive task. This hypothesis is in line with Karakas et al.'s [163] results showing that the gamma response in the 100 ms after stimulations (in different tasks) has a sensory origin, independent of cognitive tasks. Therefore, in our study, the gamma differences observed in parietal cortices could be attributed directly to the sensory and noncognitive components connected to the VWM task. This result is partially in line with findings supporting consistent variations in gamma activity in relation to memory loads [146] and promotes the hypothesis that parietal regions are part of a network of brain areas that mediate short-term storage and retrieval of phonologically coded verbal material [164]. One might propose that, for both modalities, stimuli appear to be processed in essentially the same regions during the verbal WM task. This idea, as observed by Crottaz-Herbette and colleagues [6], is consistent with the Baddeley model of WM [5, 165, 166], which proposed that both visual and auditory-verbal stimuli are translated into a code stored and manipulated in the phonological loop. This interpretation is further supported by our finding of no significant differences in gamma activation for different modality stimulation in either the Broca area (i.e., language region) or temporal areas (i.e., auditory-verbal regions), which are active during the processing of visual or auditory-verbal representation, respectively [167].

Emotions are omnipresent in human life. Learning is strictly related to emotions [168], and cognitive processes are greatly intertwined with emotional states [169]. Research on emotion and WM has focused primarily on adult clinical populations [170], but some studies investigated emotion-cognition interaction in both clinical and nonclinical populations across development [171, 172]. Other studies have demonstrated that the association between negative affect and academic performance in school is mediated [173] or moderated by WM functioning [174]. Chaouachi and colleagues [175], in order to study the learner's affective changes on the value of EI, found that emotional states are strongly correlated with the learner's EI; therefore, the evaluation of EI may facilitate in-depth investigation of the eventual impact that affective changes have on cognitive processes [175]. Our results showed that EI values decrease for each level of difficulty (from 2 to 0-back). Interestingly, this trend is inversely correlated to behavioral performance data (RTs) (Figure 8). Although the statistical analysis did not reveal a strong correlation between performance and EI, our result is in line with the above-mentioned study by Chaouachi et al. [175] that demonstrates the validity of EI as an indicator of learner performance and suggests the effectiveness of EI also in memory tasks involving children. Thus, considering that also the emotional factor is crucial in learning, we can speculate that, in a pedagogical intervention strategy aimed at optimizing the WM process, mental engagement should be taken into account in addition to other behavioral performance indicators. The absence of a statistically significant impact of the sensorial modality on EI could be an important factor in the development of pedagogical intervention aimed at enhancement of cognitive functions even in clinical populations with sensorial deficits.

## 5. Conclusion

Our findings were consistent with our predictions. Our hypothesis for the identification of an a-modal neural mediation of the theoretical phonological loop which underpins auditory-visual VWM is comprehensively supported. Specifically, the results confirm our double expectations:

1. Although the same brain areas appear to be involved in both auditory and visual VWM, there were no significant differences in the activation of neural signals with the two modalities, suggesting cross-modal processing of VWM in children.
2. The strongest significant differences in EEG activation in responses to auditory and visual n-back WM tasks depend on memory load variation. Moreover, the correlation between EI and RT results suggests how the simultaneous study of physiological and behavioral variables related to VWM could be an effective tool to enhance learning in children.

To the best of our knowledge, the present study is the first attempt to identify a neurophysiological benchmark of auditory and visual VWM in healthy children, and the results pave the way to the understanding of fine sensory influences on VWM. However, the present study is not without limitations, like the size of the sample analyzed and the average age of the participants, which means that the results could not be generalized to an older population. Moreover, we are aware that the use of 20 EEG channels cannot allow the precise indication of the areas corresponding to the activation detected at the selected 20 electrodes. Further studies on larger populations and subjects with particular clinical and sensorial conditions could contribute to the identification of eventual specific deficits and to the elaboration of training for target enhancement of WM development in childhood. Finally, the use of an experimental setup with more than 20 EEG channels could offer further developments to these first results.

## Figures and Tables

**Table 1:** Descriptive statistics of behavioral results.

		MEAN (±d.s.)			
		Load			
		Modality	*n* − 0	*n* − 1	*n* − 2
Behavioral variables	RTs	Audio	646.417 (±231.639)	674.638 (±3.3.793)	874.047 (±202.921)
		Video	468.303 (±228.142)	564.106 (±242.782)	762.179 (±282.728)
	ACC	Audio	0.969 (±0.070)	0.876 (±0.131)	0.870 (±0.108)
		Video	0.909 (±0.166)	0.833 (±0.215)	0.870 (±0.168)
	IES	Audio	664.880 (±218.066)	836.421 (±474.344)	1021.001 (±346.921)
		Video	516.207 (±180.57)	732.827 (±361.078)	1130.646 (±485.263)

**Table 2:** ANOVA analysis of behavioral results.

	ANOVA								
	Load			Modality			Load × Modality		
	*F*	*p*	*η*_*p*_^2^	*F*	*p*	*η*_*p*_^2^	*F*	*p*	*η*_*p*_^2^
RTs	25.038	**<0.001**	0.714	10.744	**<0.008**	0.517	1.285	0.298	0.113
ACC	11.7	**<0.001**	0.539	3.036	0.112	0.232	2.189	0.138	0.179
IES	17.671	**<0.001**	0.638	0.371	0.555	0.035	3.421	0.052	0.254

**Table 3:** Neurophysiological results of ANOVA analysis.

		Electrode clusters	EEG bands	Load			Modality			Load × Modality		
				*F*	*p*	*η*_*p*_^2^	*F*	*p*	*η*_*p*_^2^	*F*	*p*	*η*_*p*_^2^
Brain areas	Frontal	F3, F4, F7, F8, Fz	Theta	4.6571	**0.021**	0.317	0.672	0.431	0.063	2.273	0.128	0.185
	Midline	Fz, Cz, Pz	Theta	7.697	**0.003**	0.434	1.11	0.316	0.099	2.415	0.114	0.194
	Left Hemisphere	F3, C3, T7, P3, O1	Theta	7.624	**0.003**	0.432	0.016	0.901	0.001	1.958	0.167	0.163
	Parietal	Pz, P3, P7, P8	Gamma	2.021	0.158	0.168	0.018	0.895	0.001	5.851	**0.009**	0.369
	Engagement Index (EI)	Fz, F3, F4, F7, F8, Cz, C3, C4, T7, T8, Pz, P3, P4, P7, P8, Cp5, CP6, O1, O2	Beta/(Alpha+Theta)	8.674	**0.001**	0.464	0.116	0.74	0.011	0.937	0.408	0.085

## Data Availability

The raw data supporting the conclusions of this article and the material will be made available by the authors without undue reservation. None of the experiments was preregistered.
